# Mechanical Properties of Biocomposites Using Polypropylene and Sesame Oil Cake

**DOI:** 10.3390/polym13101602

**Published:** 2021-05-15

**Authors:** Ju-Heon Lee, Dong Hwi Kim, Youngjae Ryu, Kwan Hoon Kim, Seong Ho Jeong, Tae Yang Kim, Sung Woon Cha

**Affiliations:** Department of Mechanical Engineering, Yonsei University, 50, Yonsei-ro, Seodaemun-gu, Seoul 03722, Korea; wngjs1009@yonsei.ac.kr (J.-H.L.); donghwi.kim@yonsei.ac.kr (D.H.K.); yjryu1027@yonsei.ac.kr (Y.R.); kimkevin99@yonsei.ac.kr (K.H.K.); 93noone@yonsei.ac.kr (S.H.J.); solemio2020@yonsei.ac.kr (T.Y.K.)

**Keywords:** sesame oil cakes, polypropylene, biocomposites, extrusion process, injection molding process, mechanical properties

## Abstract

Sesame oil cakes (SOC) produced during sesame oil production can be classified as plant residues. This study aims to use SOC as a composite material for injection molding. A biocomposite containing polypropylene (PP) and SOC, namely PP/SOC, was developed and its mechanical properties were evaluated. PP/SOC is largely divided into Homo-PP/SOC (HPS) based on Homo-PP and Block-PP/SOC (BPS) based on block-PP. The specimens containing 0–50 wt% SOC were prepared through extrusion and injection molding. As a result of the evaluation, SOC acted as a reinforcement in the matrix, and HPS and BPS showed improved flexural modulus by 36.4% and 37.3% compared to the neat PP, respectively. Tensile strength, on the other hand, decreased by 58% and 55.1%, respectively. To analyze the cause of this, cross-section observation was conducted through scanning electron microscope (SEM), and phase separation and voids were confirmed to be the cause of this. Impact strength of PP/SOC tended to vary depending on the type of matrix. HPS increased by 30.9% compared to neat PP, and BPS decreased by 25%. This tendency difference appears to be the result of SOC inhibiting crystallization of PP, and it has been confirmed through x ray diffraction (XRD) and differential scanning calorimetry (DSC) analysis. Moreover, PP/SOC can be manufactured at a low cost and is environmentally friendly because it utilizes SOC, a plant residue. It can also be applied to commercial products, such as food packaging, owing to its good moldability and improved mechanical properties.

## 1. Introduction

Plastics exhibit excellent mechanical properties, productivity, and plasticity, and are used in many fields. Plastic is widely used in disposal products and its output is increasing with increasing human population. However, plastic is oil-based and difficult to dispose and decompose, thereby causing global environmental pollution problems [1,2,3].

Several studies have been conducted with the aim of solving the environmental pollution problems caused by plastics. Biocomposites, which are a combination of biomass and conventional resins, have been developed and studied as alternatives to existing oil-based plastics to address environmental pollution problems [4,5].

Lignocellulosic biomass refers to biomass containing crude fibers such as cellulose, hemicellulose, and lignin, and is used as the main raw material for biocomposites. Typical lignocellulosic biomass includes wood flour, hemp, kenaf, jute, flax, and sisal [6]. Madyan et al. studied polylactic acid (PLA)-based biocomposites that are eco-friendly and can be used for low-cost manufacturing with wood fiber as a reinforcement [7]. Cui et al. used wood sawdust as a biocomposite for high density polyethylene (HDPE). HDPE exhibits an improved flexural modulus with an increase in wood sawdust content and demonstrates an economic advantage by utilizing discarded residues [8]. Sisal fibers contain rich crude fiber, which can be used to strengthen their mechanical properties. They are priced at only one-ninth the price of synthetic fibers making them economical [9]. Joseph et al. studied the tensile properties of biocomposites using polyethylene (PE) and sisal fibers and evaluated them based on the content, shape, and orientation of the fiber [10]. Joo et al. proposed a composite pellet using spent coffee grounds (SCG) to reduce the social cost of byproducts and confirm that SCG improves the impact strength of biocomposites [11]. To this end, the present study proposes a biocomposite for injection molding utilizing sesame oil cakes (SOC) as biomass.

Sesame seeds are one of the main oil crops produced in Sudan, Burma, India, China, and South Korea. As of 2018, the global sesame seed production reached 5,937,645 tons, increasing annually [12]. During oil extraction from sesame seeds, SOC, a plant residue, is produced. The typical oil extraction rate is 50%, resulting in 50% SOC [13]. As of 2018, the global sesame oil production reached 1,059,146 tons, resulting in a large amount of SOC [12]. Chemical composition of SOC was analyzed by association of official analytical chemists (AOAC) standard method. SOC was found to consist of 5.71% moisture, 30.3% lipid, 23.7% protein, 6.12% ash, 10.8% crude fiber, and 28.8% carbohydrates [14] SOC can be recycled as animal feed because it contains abundant protein; however, this requires separate facilities and SOC cannot be used nutritionally as a standalone product [15,16]. Therefore, a more efficient recycling method is required. In this study, we propose a new method for utilizing SOC as a biocomposite for injection molding. During the oil extraction process, SOC removes most of the moisture in the material but retains the crude fiber (10.8 g/100 g), which potentially can be used as a reinforcement [14]. SOC can also maximize the economic and environmental benefits by utilizing discarded residues.

Nascimento et al. conducted a study on extruded foods using SOC. Corn grits, coarsely ground corn, were molded into extrudates using extruders, and semi-defatted sesame cakes (SDSC) was added to enhance the nutrition and sensorial characteristics of extrudates. SDSC-corn exudates showed four times higher protein content than non-SDSC-corn exudates. This is because SDSC contains high protein, and the texture improvement, such as crispness, has also been confirmed by the crude fibers contained [17]. Machado et al. developed a biocomposite using SOC in cassava starch-based foam. Foams with an SOC content of 0–40 wt% were produced using a stirrer and baking machine, and the characteristics of the SOC content were evaluated. High lipids and proteins in SOC can reduce the moisture sensitivity of composite materials. Proteins can also be rearranged by heat and force during the process, reducing the fragility of composite materials. The evaluation showed a 46% reduction in the water absorption capacity in comparison with that of neat cassava starch and most of the mechanical properties were similar to those of common expanded polystyrene (EPS) [15]. Ray et al. invented a biocomposite using sesame husks (SH), a byproduct of dehulling the sesame seed, as a filler. Unsaturated polyester resin was used as the matrix and the specimens were molded through compression molding. To improve the mechanical properties of the biocomposite, an alkali pretreatment process was performed on SH and its effectiveness was evaluated. Observations of microstructures using scanning electron microscope (SEM) showed that many holes were held, and these holes increased in number when subjected to alkali pretreatment. These holes strengthen the binding of the resin and absorb the impact, so alkali treated sesame husks (ATSH) composite has higher mechanical properties than untreated sesame husks (USH) composite. Compared to USH composite, ATSH composite showed 40% improved flexural modulus and 46% improved impact strength [18]. Wu et al. developed PTT-g-GMA/SH, a biocomposite of SH and glycidyl methacrylate-grafted polymethylene terephthalate (PTT-g-GMA), which was molded into a hot press to evaluate its mechanical and biodegradable properties. Hydroxyl groups of SH and glycidyl methacrylate groups of PTT-g-GMA combine to improve mechanical properties, especially tensile strength. SH is also a natural material, so it can improve biodegradability of composite materials. PTT-g-GMA/SH showed a tensile strength of 2–26 MPa, higher than PTT/SH, a biocomposite of SH and polymethylene terephthalate (PTT). Also, biocomposites containing 40% SH exhibited a weight loss of more than 60% after 120 days owing to biodegradation, which improved as the SH content increased [19].

However, thus far, no study has focused on utilizing SOC as a biocomposite for injection molding. The present study aims to confirm that this material can be used as a commercial product. PP/SOC, a biocomposite containing polypropylene (PP) and SOC, was developed, and its crystallinity, thermal and mechanical properties were evaluated. In addition, a SEM image of the cross-section of the specimen was observed to evaluate the SOC particle form and its binding to the matrix. PP/SOC pellets for injection molding were produced through an extrusion process and PP/SOC specimens for evaluation of each property were injection-molded. The material was intended to be applied to food packaging, which is the main use of biocomposites, and this study intends to contribute to the development of a new recycling method for SOC and eco-friendly materials.

## 2. Materials and Methods

### 2.1. Materials

PP, one of the main materials in food packaging, was used as the matrix [20]. Using two PPs, we compared the characteristics of biocomposites according to the matrix. The matrices included homo-polypropylene (Homo-PP, J-150, Lotte Chemical Corp., Seoul, Korea) and block-polypropylene (block-PP, J-350, Lotte Chemical Corp., Seoul, Korea). The melting point (Tm) and the melt flow index of the two PPs were the same at 160 °C and 10 g/10 min, respectively. Homo-PP is a semi-crystalline polymer that is polymerized only by propylene and corresponds to the most common form of PP. In contrast, block-PP is a copolymer of propylene and ethylene, and contains ethylene-propylene rubbers, resulting in lower crystallinity and higher impact strength than those of homo-PP [21].

Maleic anhydride-grafted polypropylene (MAPP, G-3003, Eastman Inc., Kingsport, TX, USA) was used as a compatibilizer. MAPP is one of the most common commercial compatibilizer [22]. PP is a non-polar hydrophobic material, and SOC is a polar hydrophilic material with hydroxyl groups [15,23]. Consequently, the interfacial adhesion between the two materials is very poor, resulting in a drop in mechanical properties. In other words, PP/SOC corresponds to a composite material, not a grafted material. In this study, we used it to check if there is a difference in mechanical properties of PP/SOC.

**Figure 1:**
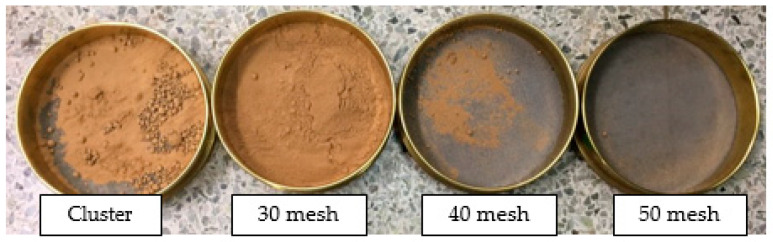
SOC particle size classification results.

The SOC in this study was supplied by a local oil mill (Sinsonsuccbulgin, Seoul, Korea). SOC collected and used 90% of China’s sesame seeds and 10% of South Korea’s sesame seeds within 2 weeks of heat compression. The SOC was dried for 24 h in an oven at a temperature of 90 °C to remove residual moisture before being used as biomass. The dried SOC was processed in powder form using a grinder (HMF-3160S, Hanilelec, Seoul, Korea). The crushed SOC powders were classified by particle size to reproduce constant properties and increase the reliability of the experiment. At this time, the sieve test machine used a self-made product. The machine consisted of seven test sieves (210(Φ) × 60(h), Chunggye Sieve Inc., Seoul, Korea). The powder was classified by particle size through left and right repetitive motions. SOC powder (100 g) was classified according to particle size (Figure 1). With 120 cycles per minute, it lasted 5 min, and on classification, the cluster SOC powder accounted for 32.31%, 30-mesh SOC powder accounted for 64.16%, and 40-mesh SOC powder accounted for only 3.53% (Table 1). In this study, 30 and 40 mesh SOC powder were used, corresponding to a particle size of 600–425 μm and 425–300 μm, respectively.

### 2.2. Extrusion Process

To evaluate the properties of PP/SOC, PP/SOC pellets for injection molding were produced. The pellet production was achieved through the extrusion process using an extrusion machine (7.5 kW, maximum RPM = 1740, Yonsei University, Seoul, Korea), conveyor belt, and cutting machine. Heaters for the extruder are installed in a total of five locations. The closer to die, the higher the temperature of the process (Table 2). When processing at a temperature of 160 °C, which is the Tm of PP, some PPs did not dissolve, making it difficult to form. relatively easy molding was possible at 170 °C. To minimize the thermal decomposition of the fibers in the SOC, molding was performed at a minimum temperature that could be processed easily [24]. The extruded PP/SOC was transferred to the cutting machine by a conveyor belt and air cooled by a fan mounted on top of the conveyor belt. Typically, extrudates are cooled by a water cooling method; however, in this study, the air cooling method was adopted considering the hydrophilic substances in SOC [25]. The cooled PP/SOC was pelletized to a length of approximately 1 cm using the cutting machine.

### 2.3. Injection Modling Process

The pelletized PP/SOC was dried at 90 °C for 12 h in an oven to remove residual moisture before injection molding. Injection molding machines (SELEX-120, L/D = 20:1, 120 tons, WOOJIN PLAIMM Corp., Korea, Boeun) were used. Two molds were used to mold the specimens. Using PP/SOC pellets, specimens with SOC 0–50 wt% were molded. A total of six heaters were installed in the injection machine, and the closer to the end nozzle, the higher the temperature. The injection process was conducted at higher temperatures than that of the extrusion process because higher flowability was required compared with that in the extrusion process (Table 3). The temperature was set to below 200 °C to minimize the degradation of mechanical properties due to thermal decomposition [24].

### 2.4. Sample Specifications

**Figure 2:**
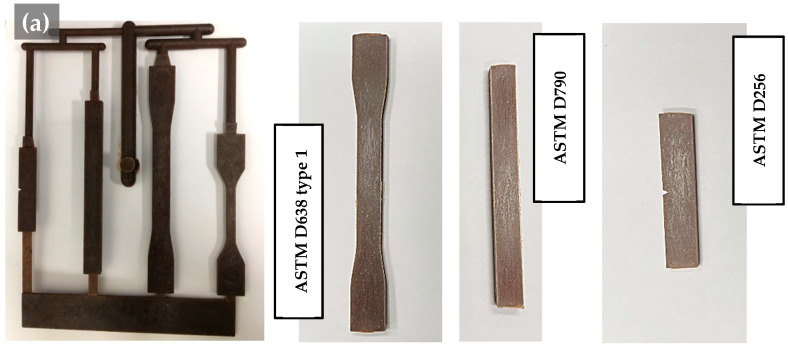

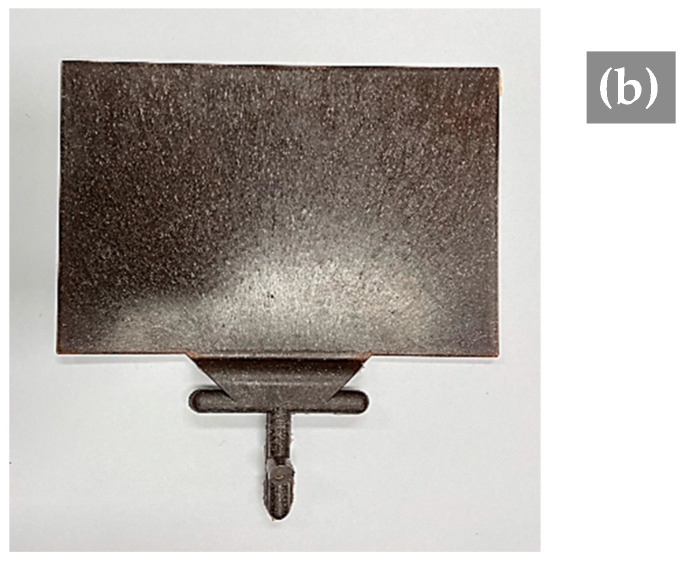
Injection-molded PP/SOC, biocomposite containing polypropylene (PP) and SOC: (**a**) specimen for mechanical properties evaluation; (**b**) specimen for SEM, XRD.

In this experiment, PP/SOC specimens with a total of 14 composition were molded (Table 4). It is largely classified as Homo-PP/SOC (HPS) based on Homo-PP and Block-PP/SOC (BPS) based on Block-PP. The characteristic differences according to SOC content were evaluated through HPP, HPS10-50, BPP, and BPS10-50. HPSm30 was used separately to determine differences in properties depending on the addition of the compatibilizer by adding 3 per hundred resin (phr) MAPP. The HPS30s used 40mesh of SOC to determine differences in characteristics depending on the particle size of SOC. Specimens of each composition were produced in two forms. The first specimen was used for evaluating the mechanical properties. It consisted of specimens based on American Society Testing and Materials (ASTM) D638 types 1, D790, and D256, each with a size of 165 × 19 × 3.3 mm^3^, 127 × 12.7 × 6.4 mm^3^, and 63.5 × 12.7 × 3.0 mm^3^ (Figure 2) [26,27,28]. The second specimen was used for SEM imaging, x-ray diffraction (XRD) and differential scanning calorimetry (DSC) analysis. Its dimensions were 150 × 100 × 1.8 mm^3^ and it was composed of thin sheets (Figure 2). XRD analysis could be performed only on samples with a powder size of less than 10 μm or thin sheets. As PP-based composite materials were difficult to process into the powder form, sheet-type specimens were separately produced and evaluated. SEM photography and DSC analysis of the relevant specimen was performed because it was easily breakable and a good section of it could be obtained.

### 2.5. Test Methods

The crystallinity of the SOC powders and PP/SOC specimens was measured using high resolution x-ray diffractometer (HR-XRD, Smartlab, Rigaku Corp., Tokyo, Japan). Cu Kα (λ = 1.5406 Å) was used as the x-ray source and the measured range of 2θ was 5–60°. The scanning speed was 2°/min, and the diffraction patterns obtained were evaluated for relative crystallization (Xc) using Origin PRO (OriginLab Corp., Northampton, PA, USA) software. Xc is the ratio of the area of the crystal peak (Ac) to the area of the total peak (At). To check the difference in the characteristics of PP/SOC according to the presence or absence of SOC, SOC powders, specimens with an SOC content of 0% and 30% were evaluated.

(1) $$X_{c}=\frac{A_{c}}{A_{t}}$$

DSC (DSC25, TA Instruments Inc., New Castle, DE, USA) analysis was performed to evaluate thermal properties of PP/SOC. It was heated and cooled in the range of 0–240 °C and proceeded at the same speed as 10 °C/min. to evaluate differences in properties with or without SOC, specimens with an SOC content of 0% and 30% were used for the measurement.

The tensile strength, flexural modulus, and impact strength of the specimens were measured to evaluate their mechanical properties. For tensile strength measurement, the specimens were stretched at a speed of 50 mm/min using a universal testing machine (UTM, QM-100T, QMESYS Co. Ltd., Seoul, Korea) until breakage within 5 min. For flexural modulus measurement, a three-point bending test was performed using UTM. The support distance was set to 102.4 mm and the measurement was performed at a speed of 20 mm/min. For impact strength measurement, the specimens were broken using a digital impact tester (ST-120, SALT Co. Ltd., Korea, Incheon), and the impact strength was evaluated based on the energy absorbed by the specimen. Mechanical properties were evaluated for specimens with an SOC content of 0–50%, and after measuring seven specimens for each condition, the average value of five specimens was calculated, excluding the maximum and minimum values.

SEM (JSM-7001F, JEOL Ltd., Tokyo, Japan) was used to observe the cross-section of the PP/SOC specimen. The specimens with an SOC content of 10–30% were cooled to liquid nitrogen and broken to obtain a fine cross-section. The specimens were coated with platinum via vacuum deposition and filmed.

## 3. Results and Discussion

### 3.1. Crystallinity

**Figure 3:**
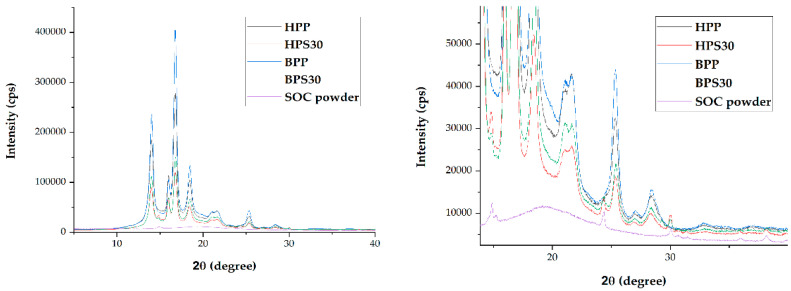
X ray diffraction (XRD) result of SOC powder and PP/SOC specimens.

XRD analysis of the SOC powder and PP/SOC specimens was performed. The crystal peak of PP is found between 10° and 30°, consisting mainly of alpha and beta crystals. There are a total of four crystal peaks of PP that stand out from the XRD results of this experiment. These include peaks of 14.02°, 15.98°, 16.78°, and 18.46°. According to a precedent study, each of these is α(110), β(300), α(040), and α (130) crystal [29]. Owing to the addition of SOC, these PP crystal peak intensities were reduced. Also, five crystal peaks were observed due to SOC (Figure 3). This corresponded to 14.88°, 24.44°, 30°, 35.97°, and 38.15°, respectively, compared to the XRD results for SOC powder. Owing to the addition of SOC, both specimens demonstrated reduced crystallinity. HPS30 showed a 7% decrease in crystallization compared to HPP, and BPS30 showed a 14% decrease in crystallization compared to BPP. This may be attributed to the large amount of amorphous materials in the SOC. The SOC powder exhibited a crystallinity of 29.11%, which is significantly low compared to that of neat PP (Table 5). SOC was also observed to inhibit the crystallization of PP. Previous studies have demonstrated that the high-volume addition of biomass reduces crystallinity by inhibiting the movement of the polymer chain [30].

### 3.2. Thermal Properties

**Figure 4:**
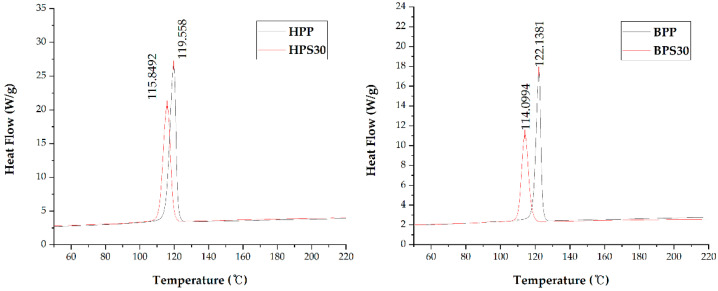
Cooling curve of differential scanning calorimetry (DSC) results.

**Figure 5:**
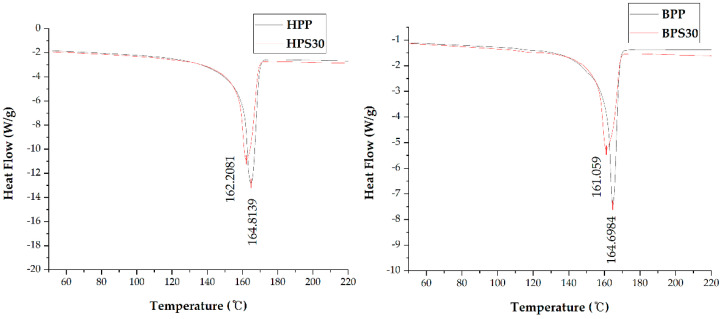
Heating curve of DSC results.

DSC analysis of PP/SOC specimens was performed. An exothermic transition by crystallization occurred in the cooling curve, where the peak was shifted to a lower temperature by the addition of SOC (Figure 4). The peak temperature corresponds to the crystallization temperature (Tc). The HPS30 showed a 3.1% reduction in Tc compared to the HPP, and the BPS30 showed a 6.6% decrease in crystallization temperature compared to the BPP. The reduction in Tc confirms that SOC acts as an inhibitor that inhibits the crystallization of PP. As a result of evaluating the heating curve of DSC, an endothermic transition by melting occurred, where the peak was shifted to a lower temperature by the addition of SOC (Figure 5). The peak temperature corresponds to the Tm. Due to the addition of SOC, HPS30 was found to have reduced melting point by 1.6% compared to HPP and BPS30 by 2.2% compared to BPP.

### 3.3. Mechanical Properties

The tensile strength, flexural modulus, and impact strength of PP/SOC were evaluated according to composition of specimens.

**Figure 6:**
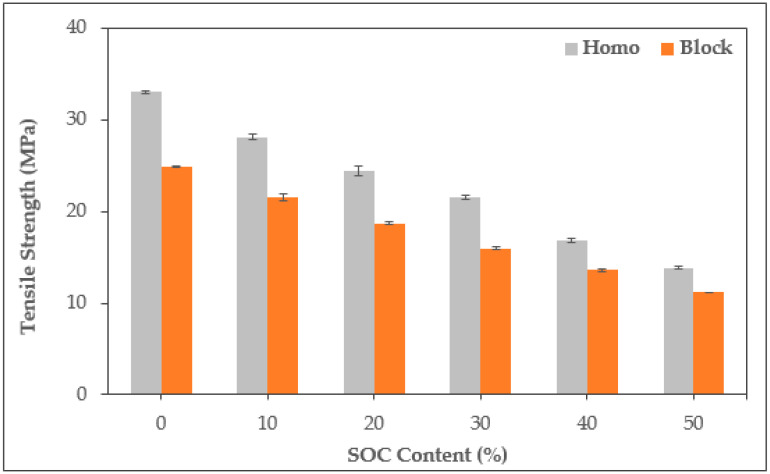
Tensile strength of PP/SOC specimen.

The tensile strength tended to decrease as the SOC content increased (Figure 6). This trend appeared in all the specimens. HPS50’s tensile strength was reduced by 58% compared to HPP, and BPS50 was reduced by 55.1% compared to BPP. This trend appears to be mainly due to the occurrence of irregular voids caused by moisture in the SOC and the phase separation between PP and SOC. SOC contains a large amount of hydrophilic material, which makes it almost impossible to completely remove moisture from the material; it also has a low interfacial adhesion with the hydrophobic PP [14,31,32].

**Figure 7:**
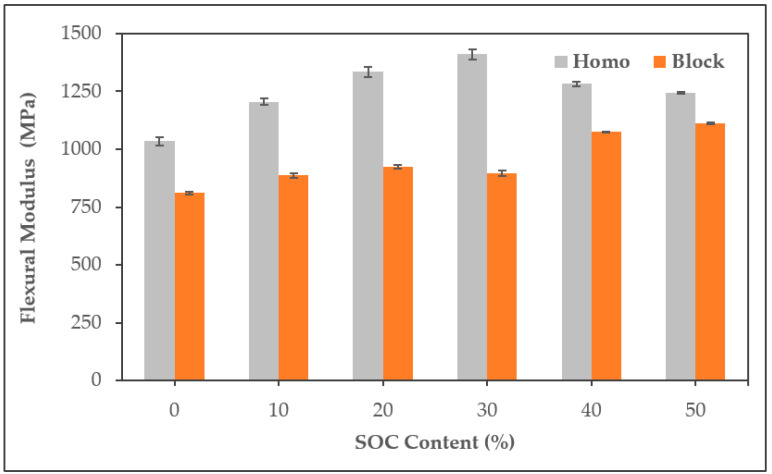
Flexural modulus of PP/SOC specimen.

PP/SOC showed improved flexural modulus compared to neat PP for all conditions. (Figure 7). Both HPS and BPS showed an improvement in modulus of up to 36.4% and 37.3%, respectively. This tends to be the opposite of the results of tensile strength and are due to the different effects of defects in materials on bending and tensile tests. In bending tests, strength is more affected by reinforcements such as fibers than weak parts such as voids [33]. Thus, the SOC appears to act as a reinforcement, resulting in the improvement of the flexural modulus under all conditions.

**Figure 8:**
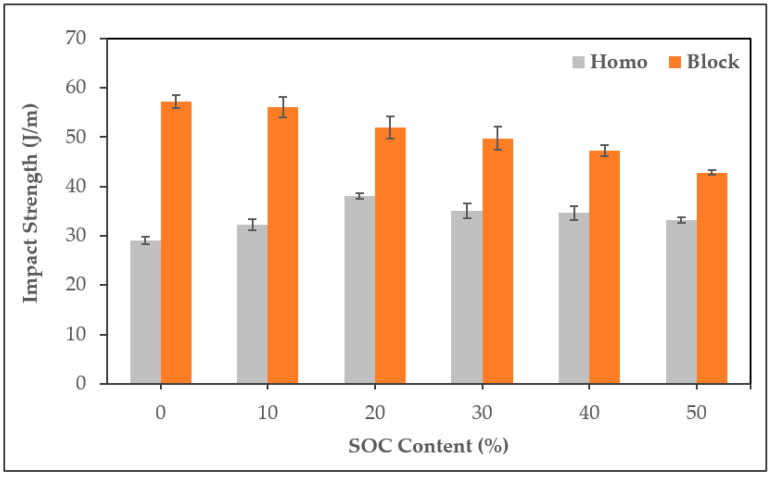
Impact strength of PP/SOC specimen.

In terms of impact strength, there was a difference in the trend depending on the matrix (Figure 8). HPS showed improved impact strength as the SOC content increased with a maximum increase of 30.9% at 20% SOC content, whereas in BPS, it declined as the SOC content increased with a maximum reduction of 25%. This trend difference has also been demonstrated in previous studies, and the difference in crystallinity between the matrix PP seems to be the reason [34]. Because of SOC, PP/SOC has a lower crystallization than that of neat PP, resulting in the improvement of the impact strength. This effect was maximized in homo-PP, a resin with high crystallinity, whereas in block-PP, the crystallinity was low for ethylene-propylene rubber, which seems to have been minimal, resulting in the opposite trend.

**Figure 9:**
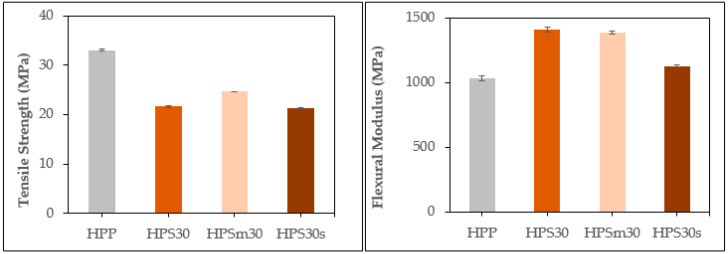

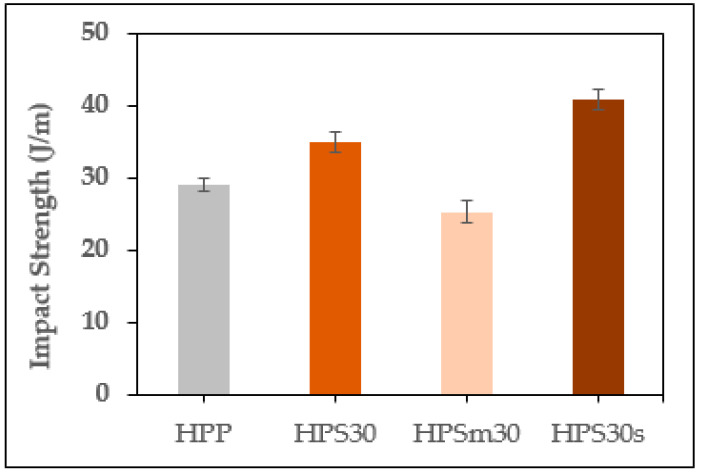
Mechanical properties of PP/SOC according to maleic anhydride-grafted polypropylene (MAPP) addition and SOC particle size.

When SOC content was 30%, we compared the mechanical properties of PP/SOC according to the addition of MAPP and particle size of SOC (Figure 9). As a result of the evaluation, the tensile strength improvement was confirmed due to the addition of MAPP. Although tensile strength was improved by 14% compared to HPS30, it showed a 25.5% decrease in strength compared to HPP and only eased the strength reduction. The flexural modulus was found to be approximately the same, with HPSm30 reduced by only 1.4% compared to HPS30. HPSm30’s impact strength decreased by 27.7% compared to HPS30. This is a 12.8% reduction compared to the HPP, in other words, a sharp drop in impact strength has been identified.

Mechanical properties of HPS30s were identified. Tensile strength was very similar to HPS30, but showed significant differences in flexural modulus and impact strength. Tensile strength decreased by1.6% but, flexural modulus was reduced by 20% compared to the HPS30. The impact strength increased by 16.8% compared to the HPS30. As the particle size of filler decreases, dramatic impact strength improvement has been shown, and this trend has also been confirmed by precedent studies [35,36,37].

### 3.4. SEM Images

**Figure 10:**
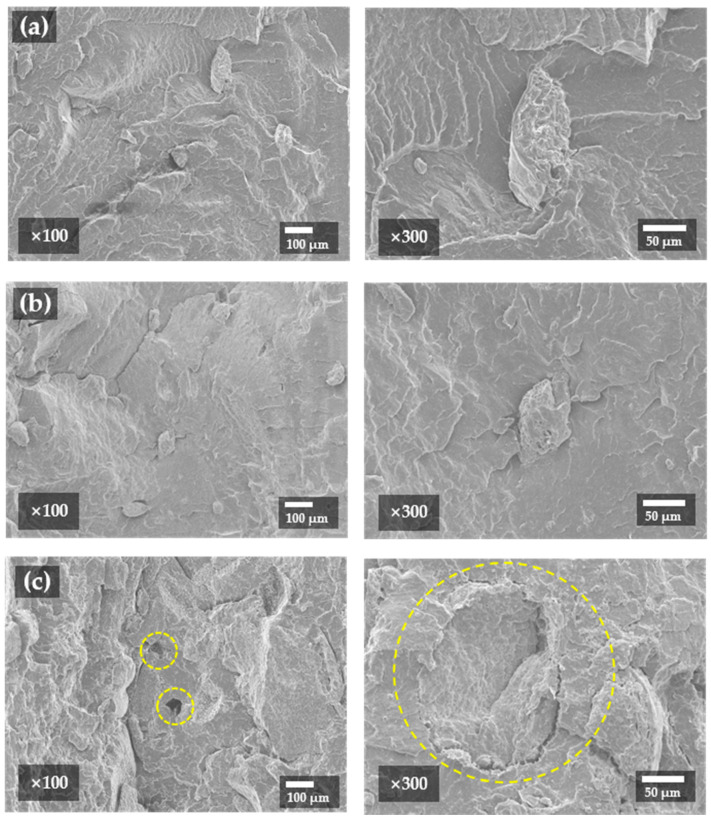
Scanning electron microscope (SEM) image of (**a**) Block-PP/SOC (BPS) with SOC 10%; (**b**) BPS with SOC 20%; and (**c**) BPS with SOC 30%.

A cross-section of the specimens with an SOC content of 10–30 wt% was observed. SOC particles in the cross-section appeared to be unstructured, and 100 μm particles smaller than 30-mesh particles were observed (Figure 10b). The SOC particles seem to have been further crushed through the extrusion and injection molding processes. Defects such as voids and phase separation were observed, which increased with increasing SOC content. The SOC content of the 30% specimen indicated that these defects were maximized (Figure 10c).

## 4. Conclusions

In this study, we developed a new method for recycling SOC as a biocomposite for injection molding. Injection-molded PP/SOC was evaluated to verify the validity of the proposed method. Prior to injection molding, PP/SOC pellets were produced through an extrusion process, and the specimens were injection-molded using them. PP/SOC exhibited good moldability in the extrusion and injection molding processes. Mechanical properties of PP/SOC were evaluated, and the cause of this was analyzed by XRD, DSC, and SEM analysis. As a result of the evaluation of mechanical properties, SOC acted as a reinforcement within PP, resulting in improved flexural modulus. Tensile strength, on the other hand, was decreased by phase separation and voids of PP and SOC. We confirm that these limitations can be overcome by using compatibilizer that can improve interfacial adhesion between PP and SOC. SOC also inhibited the crystallization of PP, resulting in improved impact strength. This has been confirmed directly through XRD and DSC, and we confirm that this effect has been maximized in HPS. SOC acts as an inhibitor to crystallization, enabling enhanced properties of PP, a semi-crystalline polymer, and PP is one of the main materials mainly used as a packaging material. Since it has excellent properties in some respects over neat PP, it can be utilized, for example, as a packaging material and has sufficient practicality due to its similar thermal stability. Furthermore, as SOC is a natural material, it can be used to develop sustainable and bio-friendly materials.

## Figures and Tables

**Table 1:** Sesame oil cake (SOC) powder particle size distribution.

SOC Powder Classification				
Mesh Number	Cluster	30	40	50
Particle Size (μm)	N/A	600	425	300
Amounts (%)	32.31 ± 1.778	64.16 ± 4.995	3.52 ± 0.341	0.01 ± 0.001

**Table 2:** Process conditions of extrusion process.

Process Conditions					
Extrusion Temp. (°C)	**Die**	**Heater 1**	**Heater 2**	**Heater 3**	**Heater 4**
	170	170	170	170	160
Die Diameter (Φ)	3				
Screw RPM	80				

**Table 3:** Process conditions of injection molding process.

Specimen Type	Specimen for Mechanical Properties			Specimen for SEM, XRD		
Injecion Temp.(°C)	**End Nozzle**	**Nozzle**	**Heater 1**	**Heater 2**	**Heater 3**	**Heater 4**
	200	200	190	180	170	160
Injection Press. (MPa)	10			5		
Injection Speed (%)	100			50		
Holding Press. (MPa)	6.7			5		
Holding Time (s)	15			5		
Cooling Time (s)	30			25		

**Table 4:** Composition of PP/SOC specimens.

Specimens	Matrix (Content (wt%))	Filler (Content (wt%))	MAPP (Content (phr))
HPP	Homo-PP (100)	-	-
HPS10	Homo-PP (90)	SOC 30 mesh (10)	-
HPS20	Homo-PP (80)	SOC 30 mesh (20)	-
HPS30	Homo-PP (70)	SOC 30 mesh (30)	-
HPS40	Homo-PP (60)	SOC 30 mesh (40)	-
HPS50	Homo-PP (50)	SOC 30 mesh (50)	-
HPSm30	Homo-PP (70)	SOC 30 mesh (30)	(3)
HPS30s	Homo-PP (70)	SOC 40 mesh (30)	-
BPP	Block-PP (100)	-	-
BPS10	Block-PP (90)	SOC 30 mesh (10)	-
BPS20	Block-PP (80)	SOC 30 mesh (20)	-
BPS30	Block-PP (70)	SOC 30 mesh (30)	-
BPS40	Block-PP (60)	SOC 30 mesh (40)	-
BPS50	Block-PP (50)	SOC 30 mesh (50)	-

**Table 5:** Relative crystallinity of SOC powder and PP/SOC specimens.

	SOC Powder	HPP	HPS30	BPP	BPS30
SOC Contents (wt%)	**-**	0	30	0	30
Xc (%)	29.11	58.51	54.41	48.61	41.59

## Data Availability

Not applicable.
